# Human papillomavirus vaccine beliefs and practice characteristics in rural and urban adolescent care providers

**DOI:** 10.1186/s12889-022-13751-3

**Published:** 2022-07-09

**Authors:** Cody L. Goessl, Ben Christianson, Kayla E. Hanson, Elizabeth J. Polter, Scott C. Olson, Thomas G. Boyce, Denise Dunn, Charnetta L. Williams, Edward A. Belongia, Huong Q. McLean, Jeffrey J. VanWormer

**Affiliations:** 1 Center for Clinical Epidemiology and Population Health, Marshfield Clinic Research Institute, 1000 North Oak Ave, Marshfield, WI 54449 USA; 2 Minnesota Department of Health, St Paul, USA; 3 U.S. Centers for Disease Control & Prevention, Atlanta, USA

**Keywords:** Human papillomavirus, Vaccination, Rural, Healthcare providers, Beliefs

## Abstract

**Background:**

The human papillomavirus (HPV) vaccine is recommended for all adolescents age 11–12 years. HPV vaccine coverage remains suboptimal in the United States though, particularly in rural areas. We surveyed adolescent immunization providers in two Midwestern states to assess rural vs. urban differences in HPV vaccine resources, practices, and attitudes.

**Methods:**

A cross-sectional survey was sent to all licensed adolescent care providers in a subset of urban and rural counties in Minnesota and Wisconsin during 2019. Multivariable regression was used to identify attitudes and practices that differentiated rural vs. urban providers.

**Results:**

There were 437 survey respondents (31% rural). Significantly fewer rural providers had evening/weekend adolescent vaccination appointments available (adjusted odds ratio (aOR) = 0.21 [95% confidence interval (CI): 0.12, 0.36]), had prior experience with adolescent vaccine quality improvement projects (aOR = 0.52 [95% CI: 0.28, 0.98]), and routinely recommended HPV vaccine during urgent/acute care visits (aOR = 0.37 [95% CI: 0.18, 0.79]). Significantly more rural providers had standing orders to administer all recommended adolescent vaccines (aOR = 2.81 [95% CI: 1.61, 4.91]) and reported giving HPV vaccine information to their patients/families before it is due (aOR = 3.10 [95% CI: 1.68, 5.71]).

**Conclusions:**

Rural vs. urban differences in provider practices were mixed in that rural providers do not implement some practices that may promote HPV vaccination, but do implement other practices that promote HPV vaccination. It remains unclear how the observed differences would affect HPV vaccine attitudes or adolescent vaccination decisions for parents in rural areas.

**Supplementary Information:**

The online version contains supplementary material available at 10.1186/s12889-022-13751-3.

## Background

Human papillomavirus (HPV) causes nearly 35,000 new cancers each year in the United States (U.S.) [1], with lifetime medical costs of conditions attributable to new HPV infections in a given year estimated at $774 million [2]. HPV vaccination, which has been available and recommended for adolescents in the U.S. since 2006 [3], could prevent 92% of HPV-associated cancer if implemented as recommended. HPV vaccine coverage, however, remains relatively low in the U.S. with 59% of adolescents having completed the HPV vaccine series in 2020 [4]. Coverage is also lower in rural areas compared to urban and suburban areas. For example, the proportion of adolescents with ≥1 HPV vaccine dose in 2020 was 68% in rural U.S. populations as compared with 78% in urban populations [4]. This is far lower coverage than for other recommended adolescent vaccinations such as tetanus, diphtheria, and acellular pertussis (Tdap) and meningococcal ACWY (MenACWY), which are currently required for school attendance in most states [5].

Reasons for the lower HPV vaccine coverage in rural areas are complex and multifaceted, including the greater influence of poverty and lower HPV vaccine literacy in rural vs. urban areas [6, 7]. Parental decisions regarding vaccination of their children have also been known to be heavily influenced by advice from their primary care provider since the HPV vaccine first became available [8, 9]. This suggests that adolescent healthcare providers in rural areas may differ from their urban counterparts on HPV vaccine resources, practices, and attitudes. There have been intimations, for example, that some providers may use less presumptive and more participatory-oriented language when recommending the HPV vaccine [10]. Other studies have found no differences between rural and urban provider perceptions about the HPV vaccine [11]. Unfortunately, there are very few studies that have directly compared rural vs. urban providers on their HPV vaccine attitudes. Using a cross-sectional survey, we compared HPV vaccine beliefs and practice characteristics for rural vs. urban adolescent immunization providers in Minnesota (MN) and Wisconsin (WI). Our objective was to use multivariable modeling to identify the HPV vaccine attitudes and practices that were most strongly associated with rural vs. urban providers.

## Methods

### Design and setting

Primary care providers who served adolescents in select rural and urban counties in both MN and WI were invited to complete a survey in 2019. The survey was administered by the Minnesota Department of Health (MDH) in MN and by the Marshfield Clinic Research Institute (MCRI) in WI.

### Participants

Lists of licensed medical providers, including medical doctors (MD), doctors of osteopathic medicine (DO), nurse practitioners (NP), and physician assistants (PA) were obtained from the Minnesota Board of Medical Practice, the Minnesota Board of Nursing, and the Wisconsin Department of Safety and Professional Services. Potentially survey-eligible providers were invited based on license status, county of practice, and specialty. All known providers in primary care specialties who provided care to children and adolescents (pediatrics, adolescent medicine, family medicine) were selected if they had an active license with an address in one of the target counties. Specialty was not available in either state for PAs, therefore all PAs with active licenses in the counties of interest were included.

**Fig. 1:**
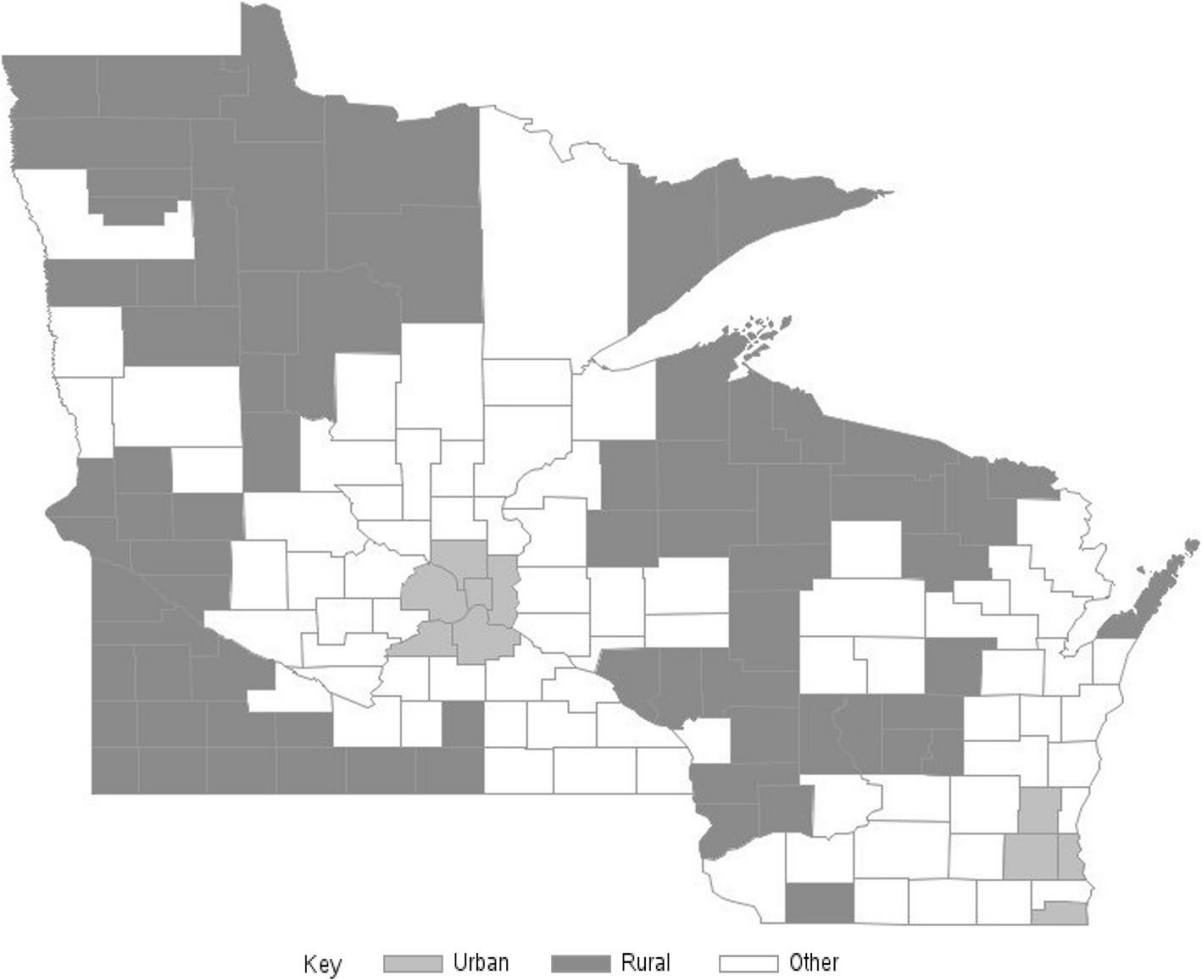
Map of study-eligible provider survey counties in Minnesota and Wisconsin, stratified by rural (dark shaded) and urban (light shaded) status based on Urban Influence Codes

All 87 counties in MN and all 72 counties in WI were initially stratified by rural and urban status based on Urban Influence Codes (UIC). The UIC system classifies counties using a 1–12 point system, with urban counties (1–2 points) based on residency size of the metropolitan area they are within (i.e., very large with ≥1 million residents or large with < 1 million residents) and the remaining non-urban counties (3–12 points) based on residency size of the largest city in the county and the county’s proximity to a metropolitan or suburban area [12]. Urban counties in this study included all those with a UIC score of 1 and with > 125,000 residents. In Wisconsin, this included the 4 counties within the metropolitan area of Milwaukee. In Minnesota, this included the 6 counties within the metropolitan areas of Minneapolis and St. Paul. Rural counties included all those with a UIC score ranging from 6 to 12 (*n* = 30 in WI, *n* = 42 in MN) (Fig. 1). This range included counties that were adjacent to small metropolitan area and with their largest city of ≥2500 residents (i.e., 6 points) to counties that were not adjacent to any metropolitan area and with their largest city/town of < 2500 residents (i.e., 12 points). Other counties with a midrange UIC score from 2 to 5 (*n* = 31 in Minnesota and *n* = 35 in Wisconsin), as well as ‘smaller’ urban counties with ≤125,000 residents (*n* = 8 in Minnesota and *n* = 3 in Wisconsin), were excluded in order to compare providers in areas that were most clearly rural vs. urban.

### Procedures

The surveys were administered through REDCap [13], with separate databases for each state. Providers with a known email address in the state database were initially invited via email to complete the survey in May–June 2019. Non-respondents received up to three additional email reminders. Remaining non-respondents, as well as providers without an email address, were mailed an invitation letter in August–September 2019. The letter included a link to complete the survey online, and a paper survey to complete and return with a prepaid mailer. Survey procedures were approved by the MCRI Institutional Review Board (IRB) with a request to waive documentation of informed consent, and were deemed exempt by the IRBs at the MDH and the U.S. Centers for Disease Control and Prevention (CDC). The study protocol was also executed in accordance with the Declaration of Helsinki.

### Measures

The survey contained 28 items on adolescent vaccine clinical resources and characteristics, as well as provider practices and attitudes, related to HPV and other adolescent vaccinations. Two initial screening questions excluded providers who did not provide preventive care, including vaccinations to adolescents aged 11–17 years, or if their primary practice setting was outside of the target counties. The first section contained questions about practice-level immunization practices and policies, such as participation in the federal Vaccines for Children (VFC) program, participation in state or regional immunization information systems or registries, standing orders for adolescent vaccinations, reminder/recall systems to notify caregivers of when adolescent vaccines are due, and vaccination hours. The section on personal vaccination practices asked providers about recommendation strength and frequency for each adolescent vaccine (Tdap, MenACWY, and HPV), including approaches to HPV vaccine recommendations and resources used to address parent/patient hesitancy. This section included several statements on how often the respondent engaged in personal vaccination practices using a 4-point Likert scale ranging from almost always to rarely/never. Items in the section on vaccine beliefs were assessed on a 5-point Likert scale ranging from strongly agree to strongly disagree. For analytical purposes, these Likert responses were categorized as yes (almost always) vs. no (frequently, occasionally, rarely/never, unknown) or agree (strongly agree, agree) vs. disagree (neither agree nor disagree, disagree, strongly disagree, unknown). The final section asked general questions about the provider and their practice setting, including specialty, years in practice, sex, practice type, panel size, proportion of patients that are adolescents, and percentage of adolescent patients on Medicaid (i.e., BadgerCare in WI and Medical Assistance or MinnesotaCare in MN).

### Analyses

T-tests and chi-square tests were used to compare general participant and practice setting characteristics. Multivariable logistic regression was used to identify attitudinal and practice exposure measures that differentiated rural vs. urban providers. The outcome was modeled dichotomously as rural vs. urban provider status. We ran a multicollinearity check between all exposures by examining their variance inflation factors [14]. As no unacceptable collinearity issues were observed, univariate models of the association between each exposure measure and rural vs. urban provider status were created first. Exposures with *p* < .10 in their univariate association were initially considered for inclusion in the final multivariable regression model. Using manual backwards selection, exposure variables were then systemically excluded until only significant (*p* < .05) determinants of rural vs. urban provider status remained in a final, reduced multivariable model. To help control for potential confounding, general characteristics that were significantly different between rural vs. urban respondents in univariate analyses, including provider specialty, number of providers in clinic, and proportion of Medicaid patients, were retained in all multivariable models. Analyses were conducted using SAS Version 9.4 (Cary, NC).

## Results

There were 5024 providers invited to complete the survey (3970 from MN and 1054 from WI), with 437 (eligible) respondents, including 344 from MN (106 rural and 238 urban) and 93 from WI (29 rural and 64 urban). Across all remaining survey invitees (*n* = 4587), 1% started but did not finish the survey, 7% started but were found to be ineligible (usually because they did not see adolescent patients), 5% had invalid contact information (invalid, duplicate, or bounce-back emails), and the remaining 86% never responded. Among respondents, 31% were rural and 79% were from MN. There was a larger percentage of rural providers who practiced family medicine. Urban providers generally worked in larger clinics, with a higher number of urban respondents who reported 10 or more providers in their clinic and more than 20 patients seen daily. A much greater proportion of rural providers (69%) reported that 25–75% of their patients were covered by Medicaid (Table 1).

**Table 1:** General Characteristics of Surveyed Adolescent Healthcare Providers in Minnesota and Wisconsin, 2019

	Rural *n* = 135	Urban *n* = 302	*p*
Provider Characteristics			
Sex			.176
Female	96 (71%)	239 (79%)	
Male	36 (27%)	57 (19%)	
Unknown	3 (2%)	6 (2%)	
License Type			
MD or DO	61 (45%)	168 (57%)	.080
PA or NP	74 (55%)	134 (44%)	
Specialty			< .001
Pediatric or adolescent medicine	20 (15%)	128 (42%)	
Family medicine	115 (85%)	174 (58%)	
Years in medical practice			.182
0–2	17 (13%)	30 (9%)	
3–9	34 (25%)	66 (22%)	
10–19	37 (27%)	73 (24%)	
20–29	28 (21%)	76 (25%)	
≥ 30	12 (9%)	49 (16%)	
Unknown	7 (5%)	8 (3%)	
Clinic Environment Characteristics			
State			
Minnesota	106 (79%)	238 (79%)	.946
Wisconsin	29 (21%)	64 (21%)	
Type of outpatient practice			< .001
Private, independent practice	9 (7%)	60 (20%)	
Health maintenance organization or network	20 (15%)	115 (38%)	
Hospital or medical center	13 (10%)	58 (19%)	
Rural health clinic	83 (61%)	2 (1%)	
Federally qualified health center	8 (6%)	41 (14%)	
Other	2 (1%)	26 (9%)	
Number of providers at practice site			.018
1–4	41 (30%)	54 (18%)	
5–10	46 (34%)	103 (34%)	
> 10	47 (35%)	143 (47%)	
Unknown	1 (1%)	2 (1%)	
Number of patients seen daily			.027
1–9	11 (8%)	28 (9%)	
10–20	106 (79%)	197 (65%)	
> 20	17 (13%)	75 (25%)	
Unknown	1 (1%)	2 (1%)	
Proportion of patients age 11–17 years			.076
Less than half	117 (87%)	229 (76%)	
Half	13 (10%)	50 (17%)	
Greater than half	2 (1%)	12 (4%)	
Unknown	3 (2%)	11 (4%)	
Proportion of adolescent patients on Medicaid			< .001
< 25%	14 (10%)	103 (34%)	
25–75%	93 (69%)	100 (33%)	
> 75%	11 (8%)	57 (19%)	
Unknown	17 (13%)	42 (14%)	

Values are reported as frequency (% of column total). *P*-values correspond to chi-square test differences between rural vs. urban providers

Of the 43 exposure variables tested in univariate models, fourteen were associated with rural provider status and were considered further in multivariable modeling (Supplemental Table). Most of these exposures were subsequently dropped in the final multivariable model, but five vaccine factors independently differentiated rural vs. urban providers, including evening/weekend appointments, standing vaccination orders, prior experience with vaccine quality improvement projects, providing HPV vaccine information before it is due, and recommending HPV vaccine during urgent care visit (Table 2). Specifically from the final multivariable model, relative to urban providers, significantly fewer rural providers had evening/weekend adolescent vaccination appointments available (unadjusted 30% rural vs. 63% urban; adjusted odds ratio [aOR] = 0.21 [95% confidence interval {CI}: 0.12, 0.36]), had prior experience with adolescent vaccine quality improvement projects (17% rural vs. 31% urban; aOR = 0.52 [95% CI: 0.28, 0.98]), and routinely recommended HPV vaccine during urgent/acute care visits (13% rural vs. 22% urban; aOR = 0.37 [95% CI: 0.18, 0.79]). In contrast, significantly more rural providers had standing orders to administer all recommended adolescent vaccines (77% rural vs. 51% urban; aOR = 2.81 [95% CI: 1.61, 4.91]) and reported giving HPV vaccine information to their patients/families before it was due (34% rural vs. 20% urban; OR = 3.10 [95% CI: 1.68, 5.71]). Other practices and attitudinal exposures were statistically similar between rural and urban providers in multivariable models. In general, adolescent vaccines were widely endorsed across respondents, but significantly more providers recommended the MenACWY (96%) and Tdap (95%) vaccine as very important compared to the HPV vaccine (89%) (*p* < 0.001).

**Table 2:** Final Multivariable Model of Vaccine Resources, Practices, and Attitudes in Rural vs. Urban Adolescent Healthcare Providers, 2019

	Rural	Urban	Multivariable adjusted odds ratio (95% CI, *p*) of rural vs. urban provider status
**Clinic environment resources**			
Evening or weekend adolescent vaccination appointments available			
Yes	41 (30%)	189 (63%)	0.21 (0.12, 0.36), *p* < .001
No or unknown	94 (70%)	113 (37%)	ref
Standing orders to administer all recommended adolescent vaccine			
Yes	104 (77%)	155 (51%)	2.81 (1.61, 4.91), *p* < .001
No or unknown	31 (23%)	147 (49%)	ref
**Personal practices**			
Worked on a prior adolescent vaccine quality improvement project			
Yes	23 (17%)	92 (31%)	0.52 (0.28, 0.98), *p* = .043
No or unknown	112 (83%)	210 (70%)	ref
Provide HPV vaccine information before it is due			
Yes	46 (34%)	60 (20%)	3.10 (1.68, 5.71), *p* < .001
No or unknown	89 (66%)	242 (80%)	ref
Almost always recommend HPV vaccine during urgent care visit			
Yes	17 (13%)	66 (22%)	0.37 (0.18, 0.79), *p* = .009
Do not do urgent care visits	6 (4%)	24 (8%)	0.38 (0.12, 1.23), *p* = .106
No	112 (83%)	212 (70%)	ref

Multivariable odds ratios adjusted for all included exposures in the final model, plus provider specialty, number of providers at their practice site, and the proportion of adolescent patients on Medicaid

## Discussion

Vaccination rates for the HPV vaccine are lower in rural parts of the U.S. [4], but few studies have directly compared rural vs. urban adolescent care providers on their vaccine resources, practices, and attitudes. As expected, our provider sample reflected many of the general differences between rural and urban primary care environments [7, 15], notably that rural practice settings are smaller and staffed mainly by family medicine practitioners. After adjustment for these factors, our findings indicate that rural practice settings had more limited evening or weekend opportunities available for adolescents to get vaccinated and, as observed or intimated in some prior studies [7, 16–18], rural providers were less likely than their urban counterparts to recommend HPV vaccine during urgent or acute care visits. In addition, fewer rural providers have participated in adolescent vaccine quality improvement programs, perhaps due to less awareness or fewer opportunities as a result of distance from large academic health or research organizations.

As this study did not assess HPV vaccine coverage at the practice level, it is unknown how care elements unique to rural providers might contribute to lower HPV vaccine coverage in rural areas. Our analyses of survey data found five significantly different attitudes/practices that differentiated rural vs. urban providers, some of which would be considered favorable elements of care in rural practices. Most notably, rural providers in our study tended to provide information about the HPV vaccine to patients/families early and before the HPV vaccine was due. In addition, and perhaps related to that point, more rural providers reported having standing orders for administration of all recommended adolescent vaccines. These factors may reflect greater needs to plan for and deliver adolescent vaccines with generally lower staffing levels and higher nurse-to-physician ratios at rural clinics [19]. These facets of rural practices are encouraging, though it is not clear from our cross-sectional design if they are a result of efforts to increase vaccination rates or if they actually help minimize disparities in HPV vaccination rates in rural areas. It remains unclear if these are more stylistic practices of rural providers or part of systematic efforts at the practice-level, but these rural practice characteristics could be a foundation for future intervention research that tests enhanced HPV vaccine communication or counseling delivered as part of primary care. If, for example, best practices [20] could be combined with increased care opportunities (e.g., evening appointments) in rural areas, it could help rural practices align closer with HPV vaccination outcomes in high performing, usually more urban, healthcare settings [21].

Most of the survey responses to questions about vaccine resources, practices, and attitudes did not significantly differ between rural and urban adolescent care providers. The clinical environment appeared to be supportive of adolescent vaccinations in general, with the vast majority of both rural and urban providers reporting most/all of their care team was aware of the importance of the HPV vaccine, as well as availability of vaccine-only appointments and the VFC program to assist low-income families. Some systems-level practices, regardless of rural or urban setting, such as reminder-recall notifications of upcoming need for vaccinations and automatic scheduling of follow-up vaccine doses [22], could be more widely implemented, as over one-third of providers did not have either of them. In addition, despite few providers (10%) reporting personal concerns about the safety of the HPV vaccine, nearly half of all rural and urban providers reported they did not feel confident they could overcome parental concerns about HPV vaccine safety, a persistent vaccine hesitancy factor [23]. Though not an issue specific to rural providers, school vaccine requirements may partially explain why more providers recommended the MenACWY and Tdap vaccines as very important relative to the HPV vaccine. Tdap is currently required for school attendance in both states and MenACWY is required in Minnesota [5].

The main study limitations include the exploratory, cross-sectional design that precluded causal conclusions, and the reliance on self-reported survey data for all exposures, which is subject to recall and self-presentation biases. Those providers who were more interested in improving HPV vaccination may have been more apt to respond, and vaccine coverage data for respondents’ patient panels was unavailable. In addition, it was not possible to distinguish providers from the same clinic, which could bias some estimates if many respondents clustered from the same practice setting. There was a relatively large volume of exposures tested and, despite no obvious collinearity concerns, could be subject to some spurious findings. For survey items covering the clinical environment, as well as personal practices and attitudes, we combined responses of ‘no’ or ‘unknown’ in order to avoid excluding missing data and to keep a consistent analytical approach across exposures. The percent of ‘unknown’ responses was generally low across items (range 0–11%), but this essentially conflates the known absence of a given exposure with what could be lack of awareness of that same exposure. A precise response rate was not possible to calculate due to limited survey eligibility information on the enumerated group of providers invited but was probably about 10%, which is low. This could be a source of selection bias if survey respondents meaningfully differ from non-respondents. Finally, our survey only captured information from providers in WI and MN, more so the latter, which limits generalizability to other parts of the U.S. and is more representative of providers in MN. With larger sample sizes, future research is also warranted to confirm our findings and examine rural vs. urban differences in provider subgroups, such as those by sex, license type (e.g., physician vs. mid-level), or specialty. In addition, larger samples would permit examination of finer strata of all providers across the rural vs. urban continuum or using different rurality classifications besides county-level UIC scores. Finally, some open-ended or interview items may also be useful in understanding contextual factors related to why providers believe HPV vaccination rates are lower in their patients.

## Conclusion

Rural disparities in HPV vaccine coverage and related supports persist [4, 24–26]. This may be fostered by at least some rural provider practices that are less conducive to routine HPV vaccination as compared to urban clinical environments, but our data suggests that most provider practices were similar in these two groups. Future research should confirm these findings in other more diverse populations and should also link provider attitudes directly to vaccination rates within patient panels. In addition, more studies are needed on social norms and parent attitudes about the HPV vaccine and how these might differ, or interact with provider practices, in rural vs. urban populations. Addressing some of the barriers to high quality adolescent healthcare in rural areas may help improve HPV vaccine coverage over time.

## Supplementary Information

**Additional file 1.**

## Data Availability

The survey data that support the study conclusions are unavailable for public access because informed consent to share said data (beyond the research team) was not obtained from study participants, but are available from the corresponding author on reasonable request.

## Abbreviations

US: United States
HPV: Human papillomavirus
MN: Minnesota
WI: Wisconsin
MDH: Minnesota Department of Health
MCRI: Marshfield Clinic Research Institute
UIC: Urban Influence Codes
MD: Medical doctor
DO: Doctor of osteopathic medicine
NP: Nurse practitioner
PA: Physician assistant
IRB: Institutional Review Board
CDC: U.S. Centers for Disease Control and Prevention
VFC: Vaccines for Children
Tdap: Tetanus, diphtheria & acellular pertussis (vaccine)
MenACWY: Meningococcal ACWY (vaccine)
aOR: Adjusted odds ratio
CI: Confidence interval
