# Supplementary material for: Human papillomavirus vaccine beliefs and practice characteristics in rural and urban adolescent care providers
Source: BMC Public Health. 2022 Jul 9;22:1322. doi: 10.1186/s12889-022-13751-3 (PMC9271237; doi:10.1186/s12889-022-13751-3)
Supplement: Supplementary file 1 — Additional file 1. [file 12889_2022_13751_MOESM1_ESM.docx]

Supplemental Table. Univariate Models and Final Multivariable Model of Vaccine Resources, Practices, and Attitudes in Rural vs. Urban Adolescent Healthcare Providers, 2019.

|  | Rural | Urban | Univariate  unadjusted odds ratio (95% CI, *p*) of rural vs. urban provider status | Multivariable  adjusted odds ratio  (95% CI, *p*) of rural vs. urban provider status |
| --- | --- | --- | --- | --- |
| **Clinic environment resources** | | | | |
| Participation in Vaccines for Children program | | | | |
| Yes | 97 (72%) | 220 (73%) | 0.95 (0.61, 1.50), p=.829 |  |
| No or unknown | 38 (28%) | 82 (27%) | ref |  |
| **Evening or weekend adolescent vaccination appointments available** | | | | |
| Yes | 41 (30%) | 189 (63%) | 0.26 (0.17, 0.40), p<.001 | **0.21 (0.12, 0.36), p<.001** |
| No or unknown | 94 (70%) | 113 (37%) | ref | ref |
| Notification system to alert adolescent patients due for vaccination | | | | |
| Yes | 86 (64%) | 171 (57%) | 1.35 (0.89, 2.04), p=.165 |  |
| No or unknown | 49 (36%) | 131 (43%) | ref |  |
| Clinic in state or regional vaccine registry | | | | |
| Yes | 127 (94%) | 289 (96%) | 0.71 (0.29, 1.77), p=.466 |  |
| No or unknown | 8 (6%) | 13 (5%) | ref |  |
| Local vaccine coordinator | | | | |
| Physician | 17 (13%) | 24 (8%) | 1.64 (0.84, 3.20), p=.150 |  |
| Nurse Practitioner/PA | 23 (17%) | 45 (15%) | 1.18 (0.67, 2.07), p=.560 |  |
| Other or unknown | 87 (64%) | 201 (67%) | ref |  |
| **Standing orders to administer all recommended adolescent vaccine** | | | | |
| Yes | 104 (77%) | 155 (51%) | 3.18 (2.00, 5.04), p<0.001 | **2.81 (1.61, 4.91), p<.001** |
| No or unknown | 31 (23%) | 147 (49%) | ref | ref |
| Vaccine-only visits available | | | | |
| Yes | 119 (88%) | 267 (88%) | 0.98 (0.52, 1.83), p=.937 |  |
| No or unknown | 16 (12%) | 35 (12%) | ref |  |
| Routine scheduling of subsequent vaccine dose appointments (after initial dose) | | | | |
| Yes | 89 (66%) | 170 (56%) | 1.50 (0.99, 2.29), p=.059 |  |
| No or unknown | 46 (34%) | 132 (44%) | ref |  |
| Proportion of care team aware of HPV vaccine recommendations and importance | | | | |
| All | 40 (30%) | 122 (40%) | ref |  |
| Most | 76 (56%) | 140 (46%) | 1.66 (1.05, 2.61), p=.029 |  |
| Few | 6 (4%) | 17 (6%) | 1.08 (0.40, 2.92), p=.885 |  |
| Unknown | 13 (10%) | 23 (8%) | 1.72 (0.80, 3.72), p=.165 |  |
| **Personal practices** | | | | |
| **Worked on a prior adolescent vaccine quality improvement project** | | | | |
| Yes | 23 (17%) | 92 (31%) | 0.50 (0.28, 0.78), p=.004 | **0.52 (0.28, 0.98), p=.043** |
| No or unknown | 112 (83%) | 210 (70%) | ref | ref |
| **Provide HPV vaccine information before it is due** | | | | |
| Yes | 46 (34%) | 60 (20%) | 2.09 (1.32, 3.28), p=.002 | **3.10 (1.68, 5.71), p<.001** |
| No or unknown | 89 (66%) | 242 (80%) | ref | ref |
| Use personal examples to help parents understand importance of HPV vaccination | | | | |
| Yes | 56 (41%) | 89 (29%) | 1.70 (1.12, 2.59), p=.014 |  |
| No or unknown | 79 (59%) | 213 (71%) | ref |  |
| Age of female adolescent patients when they receive HPV vaccination recommendation | | | | |
| 9-10 yrs | 47 (35%) | 100 (33%) | 0.94 (0.23, 3.92), p=.932 |  |
| 11-12 yrs | 78 (58%) | 185 (61%) | 0.84 (0.21, 3.46), p=.813 |  |
| 13-18 yrs | 3 (2%) | 6 (2%) | ref |  |
| Unknown | 7 (5%) | 11 (4%) | 1.27 (0.24, 6.82), p=.778 |  |
| Age of male adolescent patients when they receive HPV vaccination recommendation | | | | |
| 9-10 yrs | 44 (32%) | 88 (29%) | 1.00 (0.29, 3.50), p=.999 |  |
| 11-12 yrs | 78 (58%) | 190 (63%) | 0.82 (0.24, 2.80), p=.753 |  |
| 13-18 yrs | 4 (3%) | 8 (3%) | ref |  |
| Unknown | 9 (7%) | 16 (5%) | 1.12 (0.26, 4.80), p=.874 |  |
| Recommend HPV vaccine as very important | | | | |
| Yes | 121 (90%) | 268 (89%) | 1.10 (0.57, 2.12), p=.784 |  |
| No or unknown | 14 (10%) | 34 (11%) | ref |  |
| Recommend HPV vaccine separate from other vaccines | | | | |
| Yes | 26 (19%) | 28 (9%) | 2.33 (1.31, 4.16), p=.004 |  |
| No or unknown | 109 (81%) | 274 (91%) | ref |  |
| Recommend HPV vaccine together with other vaccines | | | | |
| Yes | 104 (77%) | 233 (77%) | 0.99 (0.61, 1.61), p=.979 |  |
| No or unknown | 31 (23%) | 69 (23%) | ref |  |
| Almost always recommend HPV vaccine during well-child care or routine health maintenance visit | | | | |
| Yes | 127 (94%) | 266 (88%) | 2.26 (0.76, 6.80), p=.144 |  |
| Do not do well-child visits | 4 (3%) | 17 (6%) | 1.12 (0.24, 5.18), p=.887 |  |
| No | 4 (3%) | 19 (6%) | ref |  |
| Almost always recommend HPV vaccine during sports physical visit | | | | |
| Yes | 106 (79%) | 233 (77%) | 1.08 (0.62, 1.89), p=.779 |  |
| Do not do sport physical visits | 8 (6%) | 19 (6%) | 1.00 (0.38, 2.65), p=.996 |  |
| No | 21 (15%) | 50 (17%) | ref |  |
| **Almost always recommend HPV vaccine during urgent care visit** | | | | |
| Yes | 17 (13%) | 66 (22%) | 0.49 (0.27, 0.87), p=.015 | **0.37 (0.18, 0.79), p=.009** |
| Do not do urgent care visits | 6 (4%) | 24 (8%) | 0.47 (0.19, 1.19), p=.112 | 0.38 (0.12, 1.23), p=.106 |
| No | 112 (83%) | 212 (70%) | ref | ref |
| Recommend MenACWY vaccine as very important | | | | |
| Yes | 130 (96%) | 292 (97%) | 0.89 (0.30, 2.66), p=.835 |  |
| No or unknown | 5 (4%) | 10 (3%) | ref |  |
| Recommend MenACWY vaccine separate from other vaccines | | | | |
| Yes | 25 (19%) | 27 (9%) | 2.32 (1.29, 4.16), p=.005 |  |
| No or unknown | 110 (81%) | 275 (91%) | ref |  |
| Recommend MenACWY vaccine during well-child care or routine health maintenance visit | | | | |
| Yes | 128 (95%) | 268 (89%) | 1.34 (0.47, 3.79), p=.585 |  |
| Do not do well-child visits | 2 (1%) | 20 (7%) | 0.28 (0.05, 1.65), p=.160 |  |
| No | 5 (4%) | 14 (5%) | ref |  |
| Recommend MenACWY vaccine during sports physical visit | | | | |
| Yes | 114 (84%) | 246 (81%) | 1.04 (0.56, 1.91), p=.910 |  |
| Do not do sport physical visits | 4 (3%) | 18 (6%) | 0.50 (0.15, 1.69), p=.263 |  |
| No | 17 (13%) | 38 (13%) | ref |  |
| Recommend MenACWY vaccine during urgent care visit | | | | |
| Yes | 20 (15%) | 66 (22%) | 0.60 (0.35, 1.05), p=.072 |  |
| Do not do urgent care visits | 4 (3%) | 15 (5%) | 0.53 (0.17, 1.64), p=.271 |  |
| No | 111 (82%) | 221 (73%) | ref |  |
| Recommend Tdap vaccine as very important | | | | |
| Yes | 131 (97%) | 285 (94%) | 1.95 (0.65, 5.92), p=.237 |  |
| No or unknown | 14 (3%) | 17 (6%) | ref |  |
| Recommend Tdap vaccine during well-child care or routine health maintenance visit | | | | |
| Yes | 123 (91%) | 268 (89%) | 0.98 (0.39, 2.47), p=.972 |  |
| Do not do well-child visits | 5 (4%) | 19 (6%) | 0.56 (0.15, 2.14), p=.399 |  |
| No | 7 (5%) | 15 (5%) | ref |  |
| Recommend Tdap vaccine during sports physical visit | | | | |
| Yes | 110 (81%) | 247 (82%) | 0.97 (0.52, 1.80), p=.921 |  |
| Do not do sport physical visits | 8 (6%) | 18 (6%) | 0.97 (0.35, 2.66), p=.949 |  |
| No | 17 (13%) | 37 (12%) | ref |  |
| Recommend Tdap vaccine during urgent care visit | | | | |
| Yes | 28 (21%) | 66 (26%) | 0.72 (0.44, 1.17), p=.184 |  |
| Do not do urgent care visits | 6 (4%) | 15 (6%) | 0.64 (0.25, 1.65), p=.353 |  |
| No | 101 (75%) | 221 (68%) | ref |  |
| Use websites to convince hesitant parents about HPV vaccine | | | | |
| Yes | 31 (23%) | 74 (25%) | 0.92 (0.57, 1.48), p=.728 |  |
| No or unknown | 104 (77%) | 228 (76%) | ref |  |
| Use videos to convince hesitant parents about HPV vaccine | | | | |
| Yes | 4 (3%) | 4 (1%) | 2.28 (0.56, 9.23), p=.250 |  |
| No or unknown | 131 (97%) | 298 (99%) | ref |  |
| Use school vaccine requirements to validate vaccine importance | | | | |
| Yes | 70 (52%) | 119 (39%) | 1.66 (1.10, 2.50), p=.016 |  |
| No or unknown | 65 (48%) | 183 (61%) | ref |  |
| Use scripted or prepared answers to common concerns or questions about HPV vaccine | | | | |
| Yes | 53 (39%) | 102 (34%) | 1.27 (0.83, 1.93), p=.269 |  |
| No or unknown | 82 (61%) | 200 (66%) | ref |  |
| Use communication strategies to address concerns | | | | |
| Yes | 55 (41%) | 112 (37%) | 1.17 (0.77, 1.77), p=.468 |  |
| No or unknown | 80 (59%) | 190 (63%) | ref |  |
| Use educational brochures for patients and/or parents | | | | |
| Yes | 94 (70%) | 190 (63%) | 1.35 (0.88, 2.09), p=.175 |  |
| No or unknown | 41 (30%) | 112 (37%) | ref |  |
| Use posters when discussing HPV vaccine with hesitant parents | | | | |
| Yes | 51 (38%) | 88 (29%) | 1.48 (0.96, 2.26), p=.074 |  |
| No or unknown | 112 (83%) | 210 (70%) | ref |  |
| Received formal communication training related to HPV vaccination | | | | |
| Yes | 58 (43%) | 134 (44%) | 0.94 (0.63, 1.42), p=.784 |  |
| No or unknown | 77 (57%) | 168 (56%) | ref |  |
| Preferred delivery format of training on HPV vaccine communication | | | | |
| At a conference | 14 (10%) | 15 (5%) | 2.25 (1.01, 5.04), p=.048 |  |
| Online | 65 (48%) | 157 (52%) | 0.99 (0.64, 1.57), p=.997 |  |
| Other or unknown | 10 (7%) | 19 (6%) | 1.27 (0.55, 2.94), p=.577 |  |
| At my practice | 46 (34%) | 111 (37%) | ref |  |
| **Attitudes** | | | | |
| Concerns regarding the safety of the HPV vaccines | | | | |
| Agree | 16 (12%) | 30 (10%) | 1.22 (0.64, 2.32), p=.547 |  |
| Disagree or unknown | 119 (88%) | 272 (90%) | ref |  |
| Able to convince hesitant parents to get the HPV vaccine | | | | |
| Agree | 86 (64%) | 186 (62%) | 1.10 (0.72, 1.67), p=.674 |  |
| Disagree or unknown | 49 (36%) | 116 (38%) | ref |  |
| Influential in parents’ final decision to get the HPV vaccine | | | | |
| Agree | 112 (83%) | 251 (83%) | 0.99 (0.58, 1.70), p=.965 |  |
| Disagree or unknown | 23 (17%) | 51 (17%) | ref |  |
| Enough time to ask parents about HPV vaccine refusal or delay | | | | |
| Agree | 76 (56%) | 157 (52%) | 1.19 (0.79, 1.79), p=.404 |  |
| Disagree or unknown | 59 (44%) | 145 (48%) | ref |  |
| Confident I can overcome parental concerns about HPV vaccine safety | | | | |
| Agree | 78 (58%) | 173 (57%) | 1.02 (0.68, 1.54), p=.923 |  |
| Disagree or unknown | 57 (42%) | 129 (43%) | ref |  |

Multivariable odds ratios adjusted for all included exposures, plus provider specialty, number of providers at their practice site, and the proportion of adolescent patients on Medicaid. Bold values were significant and retained in the final multivariable model.
